# Application of the SwissDrugDesign Online Resources in Virtual Screening

**DOI:** 10.3390/ijms20184612

**Published:** 2019-09-18

**Authors:** Antoine Daina, Vincent Zoete

**Affiliations:** 1 Molecular Modeling Group, SIB Swiss Institute of Bioinformatics, University of Lausanne, Quartier UNIL-Sorge, Bâtiment Amphipôle, CH-1015 Lausanne, Switzerland; antoine.daina@sib.swiss; 2 Department of Fundamental Oncology, University of Lausanne, Ludwig Lausanne Branch, Route de la Corniche 9A, CH-1066 Epalinges, Switzerland

**Keywords:** virtual screening, computer-aided drug design, SwissSimilarity, SwissTargetPrediction, web-based tools

## Abstract

SwissDrugDesign is an important initiative led by the Molecular Modeling Group of the SIB Swiss Institute of Bioinformatics. This project provides a collection of freely available online tools for computer-aided drug design. Some of these web-based methods, i.e., SwissSimilarity and SwissTargetPrediction, were especially developed to perform virtual screening, while others such as SwissADME, SwissDock, SwissParam and SwissBioisostere can find applications in related activities. The present review aims at providing a short description of these methods together with examples of their application in virtual screening, where SwissDrugDesign tools successfully supported the discovery of bioactive small molecules.

## 1. Introduction

Different computer-aided drug design (CADD) techniques are great assets to efficiently support experimental screening by enriching chemical collections with compounds bearing desired properties and therefore reducing the number of physical samples to be assayed [1]. The computed and predicted properties are project-dependent and generally related to the pharmacodynamics or the pharmacokinetics of the molecular structures under investigation. The pharmacodynamic properties, that is, the ability of a small molecule to be recognized by the macromolecular target at the atomic level, are the central notion of virtual screening (VS) approaches.

Structure-based virtual screening (SBVS) requires biostructural knowledge about the macromolecular target and classically consists in submitting a chemical collection to ligand–protein docking simulations towards a tridimensional structure of the target. Once reliable binding modes have been predicted, estimated binding free energies or other approximate fitness scores, quicker to calculate yet less accurate, constitute the traditional criteria to select compounds with a high probability to bind to the target of interest. Many posing algorithms and scoring functions have been developed and their comparative performance has been thoroughly reviewed [2,3,4]. For instance, the docking engine behind the SwissDock web service [5] was developed for accurate physics-based estimation of binding modes and free energies according to the full definition of the CHARMM force field [6,7]. Consequently, the calculation time is too substantial to treat large chemical libraries. However, SwissDock is useful for two-step SBVS strategies, where a first approximative docking is performed by a fast engine (e.g., AutoDock VINA [8] or FRED [9]) followed by validation of a small number of selected compounds through slower, more comprehensive methods [10,11].

Ligand-based virtual screening (LBVS) estimates the propensity of a small molecule to bind to a target based on how much it resembles known active compounds. The *similarity principle* postulates that similar small molecules are prone to have similar properties, including biological activity [12]. Although it relies on a simple concept, diverse LBVS schemes exist, differing mainly in the molecular description and hence in the way to estimate similarity [13,14]. Briefly, the molecular description can be classified into 1D, 2D or 3D. 1D-descriptors are global descriptions of molecular or physicochemical properties, such as the molecular weight for size, or the partition coefficient (log *P*) for lipophilicity. They are typically employed as first estimates of the pharmacokinetic properties of molecules. Their main application in VS is the filtering of chemical collections to build focused libraries (e.g., drug-like collections) or to account for project-specific requirements (e.g., optimal properties to cross the blood-brain barrier) [15]. To this end, web tools like SwissADME, which calculate numerous descriptors linked with pharmacokinetics models, are valuable resources [16] (refer to Section 2).

2D-descriptors are derived from two-dimensional chemical structures. Many different molecular fingerprints (FP) have been proposed [14,17] and differ in their way of mapping the existence of chemical features into a bit string. A popular example is FP2 implemented in OpenBabel [18] and derived from the pioneering topological (also called path-based) Daylight FP [19]. This technique collects every possible fragment following a linear path in the chemical structures up to a given number of bonds. The presence or absence of fragments is then hashed into a 1024-bit string. Although of great simplicity, the FP2 technique outperforms other types of more complex methods in the LBVS context [20].

3D-descriptors consider the conformation of the molecule, either explicitly, i.e., actual tridimensional geometries are overlaid in the 3D-space to evaluate similarity (as for example in ROCS [21] or Shape-IT [22]) or implicitly in the so-called non-superpositional shape-based methods as for example in the Spectrophores [23] or the ElectroShape [24] approaches. In particular, ElectroShape compresses the information of the molecular shape into a one-dimensional vector and is fast enough to be reported as highly efficient and can perform for the LBVS application [25]. In brief, the technique consists in placing centroids around the conformation and calculating the distances between them and every atom. A float vector is built with the mean, standard deviation and cubic root of three first moments of the distance distributions.

During the development of the ligand-based reverse screening engine behind the SwissTargetPrediction web tool (refer to Section 2), FP2 and ElectroShape were found to be highly complementary [26], and the similarity principle was validated for both 2D- and 3D-descriptions [27]. The current target fishing engine employs a dual quantification of similarity composed of the Tanimoto index between FP2 binary vectors (for chemical similarity) and Manhattan-based values between ES5D float vectors (for shape similarity). The latter is an ElectroShape vector, where the Cartesian coordinates of each atom are complemented with an atomic partial charge and a lipophilic contribution leading to a 18-float vector describing molecular shape, charge distribution and lipophilicity projection [25]. Those two metrics of similarity between molecules are combined by a logistic equation leading to the so-called *Combined-Score*, ranging from 0 (absolutely dissimilar compounds) to 1 (identical molecules). This Combined-Score, as well as its components (FP2 and ES5D), together with other 3D methods, are implemented in the SwissSimilarity web tool for direct LBVS [28]. 

The SIB Swiss Institute of Bioinformatics is implementing the ambitious SwissDrugDesign project, which aims at providing a comprehensive web-based in silico drug design environment, freely accessible for the scientific community worldwide. Its ultimate objective is to offer a collection of integrated tools covering all aspects of CADD.

As mentioned above, some of those tools were specifically developed for direct LBVS on already-prepared libraries by using different methods to quantify molecular similarity. Other tools can be used to perform molecular docking on the targeted macromolecule surface, to calculate pharmacokinetic-related properties, or to provide possible targets of bioactive compounds through reverse LBVS.

In this short review, the SwissDrugDesign web tools and their underlying technologies are briefly described, along with their usage in the context of virtual screening. Subsequently, we provide examples of their application in research studies that discovered therapeutically relevant agents thanks to the support of these web-based methods.

## 2. Available Resources from the SwissDrugDesign Project

**Figure 1:**
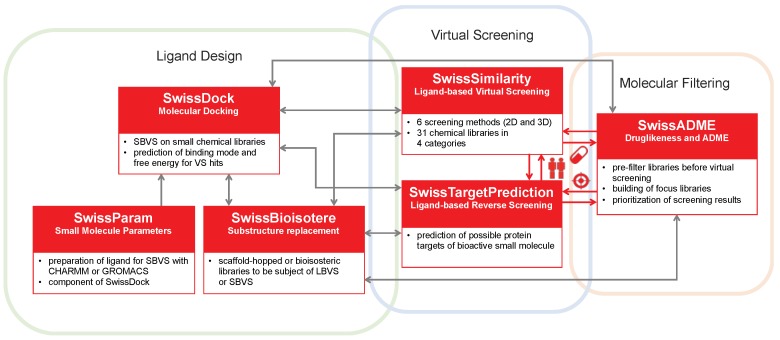
Virtual screening applications of the different on-line tools of the SwissDrugDesign project (boxed in red). Grey arrows represent “soft” relationships, for which the output of one tool can be the input of another tool by means of some user manipulation (e.g., copy/paste of SMILES). Red arrows represent actual interoperability capacities. In this way, submission of the result of one tool is simply achieved by “one-click” on the icon corresponding to the desired tool: “twins” for SwissSimilarity, “target” for SwissTargetPrediction and “pill” for SwissADME.

The SwissDrugDesign project gathers together individual on-line tools closely related either in principle or through actual links. The project itself does not refer to a website, while every component tool is reachable at its own URL or by way of interoperability features (see Figure 1). SwissDrugDesign started in 2010 with the creation of the SwissDock web-based docking engine. Since then, several other approaches have been provided to the scientific community as free websites. Of note, all tools can be accessed directly without registration and are cost-free for not-for-profit research and teaching. 

SwissDock [5] (http://www.swissdock.ch, since 2010) is a freely accessible ligand–protein docking web service and interface to predict the molecular interactions that may occur between a target protein and a small molecule. 

SwissDock is based on the EADock DSS [29] software benefiting from the most efficient features of the EADock2 algorithm [30], which is physics-based because it follows the full definition of the CHARMM force field [31]. The current version is fast enough to be operated through the web and for serial docking of small chemical libraries in the context of SBVS. Using the LPDB benchmark set of protein–ligand complexes [32], EADock DSS demonstrated a 55% success rate in reproducing the molecular interactions between proteins and drug-like molecules, when considering only the top-ranked solution over the entire protein surface (i.e., blind docking), and 64% when including the five top-ranked solutions. Docking of a LPDB complex takes 24 minutes on average on the server. 

The simplified interface has proven to drastically lower technical barriers and therefore gives access to molecular docking to a larger public than just the traditional molecular modeling community. For instance, it is possible to automatically retrieve the 3D structure of the protein from the PDB [33] and the ligand structure from the ZINC [34] databases. In a transparent way for the user, the structures of the protein and of the ligand are then automatically prepared for docking. The calculation is performed on in-house servers, and the results are displayed in 3D on the results web page for easy interpretation. On the other hand, experts can provide manually prepared protein and ligand structures and download the results for further visualization and analysis with their preferred programs. Results are stored and accessible online for 7 days.

SwissParam [6] (http://www.swissparam.ch, since 2010) is a web service that provides topologies and parameters for small organic molecules. SBVS, like other structure-based techniques for drug discovery, frequently relies on ligand–protein docking and rapid estimation of the binding free energy. This requires force field parameterization for all drug candidates. SwissParam is a fast force field generation tool, able to produce topologies and parameters based on the Merck Molecular Force Field [35] for any small tridimensional molecular structure provided in the MOL2 format. The parameters and topologies are provided in a functional form compatible with the CHARMM [36] force field. Output files can directly be used in CHARMM or GROMACS [37]. SwissParam results are extensively used by SwissDock to parameterize ligands.

SwissBioisostere [38] (http://www.swissbioisostere.ch, since 2012) was the first comprehensive and freely accessible database collecting over 4.5 million molecular substructural replacements extracted from the literature, along with information on how frequently such replacements were applied in the past, and the impact on the measured biological activity. This knowledge is of particular interest for modifying small molecules, to possibly increase affinity, or to circumvent a pharmacodynamics, pharmacokinetics, or intellectual property issue. Substitution of central cores by fragment replacements can find application in scaffold-hopping, while modification of peripheral groups can efficiently support lead optimization efforts. The output of SwissBioisostere can be employed by experts to build small chemical libraries of potential bioisosteric or related structures which can be then subject to VS. 

SwissTargetPrediction [27,39] (www.swisstargetprediction.ch, since 2014 with major updates in 2019) is a web tool aiming at predicting the most probable protein targets of bioactive small molecules. Such predictions are useful to understand the molecular mechanisms underlying a given phenotype, to rationalize possible favorable or unfavorable side effects, to predict off-targets of known molecules and to lay a rational foundation for drug repurposing. SwissTargetPrediction is a reverse LBVS method for target fishing relying both on 2D and 3D similarity measures [26] through a dual-scoring logistic regression. The prediction is provided through a user-friendly web interface for proteins of different species [40] to allow users to easily map predictions between source organisms based on target homology. In the latest version [27], SwissTargetPrediction generates predictions by reverse screening a collection of 376,342 compounds known to be experimentally active on a set of 3068 macromolecular targets. Moreover, the method estimates the probability with which the query molecule, assumed to be bioactive, will bind each predicted protein in a ranked list. It also provides the structures of the most similar active compounds (in 2D and in 3D), that drove the prediction. This latter capability is an important asset of the tool for drug design applications.

SwissADME [16] (www.swissadme.ch, since 2016) is a web tool that provides free access to a pool of fast yet robust predictive models for physicochemical properties, pharmacokinetics, drug-likeness and medicinal chemistry friendliness. SwissADME is a gateway to in-house advanced methods such as iLOGP [41] (a physics-based model for lipophilicity) or the BOILED-Egg [15] (an intuitive graphical classification model for gastrointestinal absorption and brain access). It is the first web interface that enables batch calculations for hundreds of different molecules, allowing efficient pharmacokinetic optimization as well as chemical library analysis. The latter ability is of major interest to prefilter compound collections before actual VS. This can be applied following generally accepted properties to consider, for example, only drug-like, non-toxic, stable and soluble compounds, excluding PAINS [42] or other problematic moieties [43]. Additionally, other project-specific properties relating to absorption, distribution, metabolism, excretion (ADME) or pharmacokinetics can be checked, such as, for instance, particular parameter ranges to predict optimal brain access.

SwissSimilarity [28] (http://www.swisssimilarity.ch, since 2017) is the first online, simple yet powerful LBVS tool for the rapid screening of small to very large libraries of drugs, bioactive small molecules and commercially available or virtual, yet synthesizable, compounds. Direct in silico screening can be performed using different and complementary 2D and 3D approaches to support hit finding by selecting compounds or enriching chemical collections with new molecules similar to known active ones. 

Available similarity measures are FP2 topological chemical fingerprint [18], non-superpositional Electroshape-5D [25] and Spectrophores [23] shape-based measures, Shape-IT and Align-IT (Silicos-IT [44]) tridimensional superimposition procedures, and finally the Combined-Score similarity function, as those behind the SwisstargetPrediction engine.

SwissSimilarity offers four categories of small-molecule libraries that have been prepared, ready to be screened by the above-mentioned methods:

- drug-related molecules, extracted from DrugBank [45] and further sub-divided into approved (1500 compounds), experimental (4800), investigational (500) and withdrawn drugs (160 compounds), as well as illicit (170) and nutraceutical compounds (78);
- bioactive small molecules, including, for instance, a collection of ligands found in a complex with macromolecular structures present in the Protein Data Bank (PDB) entries [33] and retrieved from LigandExpo [46] (19500 compounds), the most active molecules from ChEMBL [47] (177,000) or molecules from ChEBI [48] (28,000 compounds);
- commercially available compounds taken from ZINC [49], further sub-divided between drug-like (10,600,000 compounds), lead-like (4,300,000) and fragment-like molecules (700,000), or grouped by vendors (9,700,000 compounds);
- a collection of 205 million virtual compounds readily synthesizable from commercially available reagents using a one-step click chemistry reaction [50], and filtered for chemical stability, lack of toxicity or promiscuous characters.

**Figure 2:**
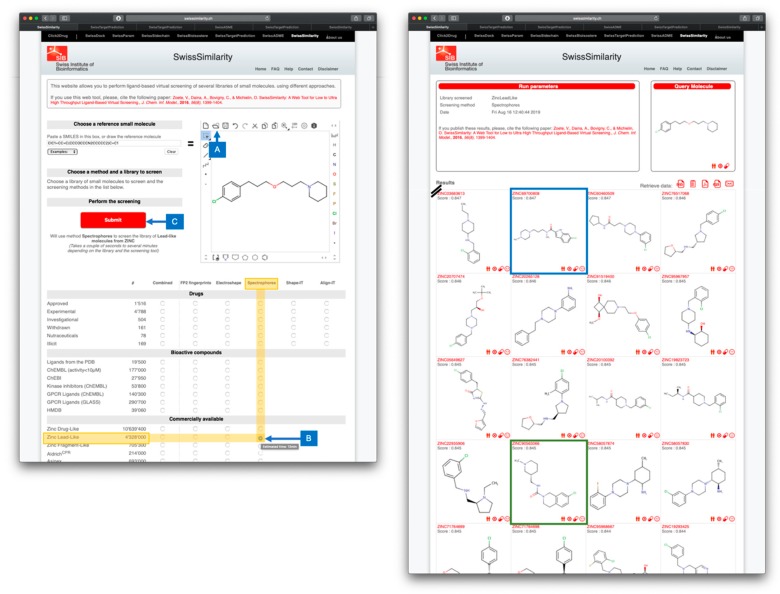
Example of LBVS by using the SwissSimilarity web tool, as in ref [57]. The left panel shows the input of the query molecule; here pitolisant can be entered by its common name, by making use of the import feature of Chemaxon Webservices (A). The method (here Spectrophores) and the database to screen (here the lead-like subset of ZINC) are boxed in yellow showing the corresponding radio button (B). Leaving the mouse over it gives an estimate of the computation time (here 12 minutes for 4,328,000 compounds). By clicking the radio button, the submission button becomes red and active (C) and the parameters selected for screening are written in full. The user can freely click on Submit to launch the calculation. The right panel displaying the result of the screening appears automatically at the end of the calculation, as another web page. The authors selected compounds ZINC69700808 (ranked #62 with a similarity score 0.847, boxed in blue) and ZINC905630066 (ranked #74 with a similarity score 0.845, boxed in green) according to subsequent analyses, including reverse LBVS with SwissTargetPrediction and pharmacokinetic parameters estimation with SwissADME. Both compounds were validated experimentally in vitro as submicromolar inhibitors of the histamine H_3_ receptor.

As for SwissBioisostere, SwissTargetPrediction and SwissADME, query molecules can be inputted in SMILES notation or drawn in the MarvinJS online sketcher, linked with Chemaxon Webservices (https://chemaxon.com). Output compounds are displayed within the web browser for easy visualization, along with their calculated similarity to the query compound. For reporting or further analysis, the output of the screening can also be downloaded as a CSV file, while the full report can be retrieved as PDF or JPEG files (for an illustration, refer to Figure 2). Importantly, interoperability between SwissDrugDesign tools is provided by dedicated buttons; each output compound can be redirected to SwissTargetPrediction, SwissADME or SwissSimilarity itself as an input for further calculations, through one click on the icon corresponding to the tool. Likewise, each compound appearing on SwissTargetPrediction and SwissADME output pages can be redirected to SwissSimilarity upon a simple click, insuring a seamless integration of these three tools. 

As an illustration of the usefulness of these tools, the foundational SwissParam and SwissDock articles have been cited 498 and 476 times since their publication in 2011, according to Clarivate Analytics Web of Science as of September 2019. More recent tools like SwissADME, SwissTargetPrediction, SwissBioisostere and SwissSimilarity have been cited 290, 123, 47 and 29 times, respectively. The web traffic to our tools reached a total of 150,000 unique visitors from more than 150 countries during the July 2018–June 2019 period. These users opened 320,000 sessions and submitted 610,000 calculations.

## 3. Examples of Applications in Virtual Screening Empowered by SwissDrugDesign Tools 

Several screening campaigns and related medicinal chemistry studies benefitted from the SwissDrugDesign collection of web-based CADD tools. 

The RAC-alpha serine/threonine-protein kinase (AKT1) and focal adhesion kinase (FAK) play key roles in normal cell signaling. However, it has been established that the AKT1–FAK interaction facilitates cancer metastasis by increasing cell adhesion under increased extracellular pressure [51,52]. Blocking the AKT1–FAK interaction is therefore an attractive rational to avoid or limit metastasis in cancer therapy. Basson and coworkers identified a seven-residues short peptide from FAK that binds AKT1 and prevents pressure-activated cancer cell adhesion [53]. They subsequently used this peptide as a reference structure to virtually screen a ZINC-based library of 10,639,555 commercially available molecules though a 3D shape-based method, using the ROCS software (OpenEye), in the search for potential AKT1–FAK inhibitors. The authors selected one compound discovered by this 3D virtual screening, tagged ZINC04085549, and used it as a query for both the SwissSimilarity web tool and the http://zinc.docking.org/search/structure search engine to look for shape or chemically similar structures in ZINC. Finally, eleven compounds were selected for an experimental assay. Two of them, i.e., ZINC04085549 (discovered using ROCS) and ZINC4085554 (identified using the Electroshape-5D approach within SwissSimilarity), were shown to prevent pressure-stimulated increases in the adhesion of a colon cancer cell model. Recently, ZINC4085554 was further confirmed to inhibit the AKT1–FAK interaction in response to increased extracellular pressure [54], suggesting a potential role of this molecule discovered through virtual screening and its chemotype in the prevention of metastasis.

With the aim of discovering new histamine H_3_ receptor (H_3_R) effectors, an important GPCR target for narcolepsy [55] also implied in the physiopathology of various metabolic and neurodegenerative diseases [56], Ghamari et al. established a comprehensive workflow combining various VS approaches using three different starting points [57]. The first one is structure-based and starts by building pharmacophores with LigandScout [58] and using them to screen the ZINC database with ZINCPharmer [59]. The second one is purely ligand-based and makes use of SwissSimilarity to find similar compounds to the commercial H_3_R inverse agonist pitolisant [55] in the drug-like set of ZINC. Independent screenings were performed following either 2D (FP2 fingerprints) or 3D (ElectroShape5D or Spectrophores) similarity measures. The third starting point is a hybrid one, involving a simple search on the ZINC website and using the structures as a small library for LigandScout. Every output compound from all three starting points was then filtered according to SwissTargetPrediction, keeping only those with sufficient probability to have H_3_R as a predicted target. At this stage, the ligand-based route generated 64 virtual hits, the structure-based route generated one virtual hit and the hybrid route generated 26 virtual hits. The next crucial step, prediction of ADME parameters, significantly reduced the number of compounds to follow-up with more demanding simulations or experiments. Indeed, by applying the drug-likeness filters of SwissADME in a consensus manner and using the BOILED-Egg model [15] to estimate blood–brain barrier (BBB) passive crossing, the number of virtual leads dropped to five molecules in total. Three out of these five compounds were validated with in vitro affinity (IC_50_) to H_3_R as low as 0.49, 0.54 and 1.2 µM for ZINC69700808 (ligand-based route), ZINC90563066 (ligand-based route) and ZINC2895674 (hybrid route), respectively.

The strategy consisting in assessing a de-risked marketed drug or a late development molecule for another medical indication, termed drug repurposing, is commonly followed nowadays [60,61,62,63]. Reverse screening target prediction, but also direct virtual screening, can play a role in laying the rational foundation in that context. As an illustration, Hassan et al. [64] employed the 2D/3D combined method of SwissSimilarity to search for molecules similar to the marketed acetylcholinesterase (AChE) inhibitor donepezil, within the collection of 1516 FDA-approved drugs. Thirty-six drugs were selected for the subsequent structure-based selection process, including consensus docking studies involving AutoDock Vina [8], AutoDock 4 [65] and GLIDE [66]. This led to a shortlist of ten drugs to undergo pharmacogenomics analysis. Cinitapride, risperidone, domperidone, tamsulosin and verapamil showed a clear bias towards Alzheimer disease-associated genes in comparison to other networks. Those five drugs were finally tested in vitro for AChE inhibition. Cinitapride showed an IC_50_ of 0.11 µM and non-competitive inhibition kinetics, displaying a favorable repurposing profile for Alzheimer disease.

Besides classical hit finding activities, LBVS has successfully been employed for various related applications. A few examples are given in the following.

PKMYT1, a membrane-associated inhibitory kinase, is an attractive research target in oncology, as its inhibition can induce relatively tumor specific apoptosis through blocking the second checkpoint G2 [67]. Schmidt and coworkers [68] applied an elegant structure-based strategy on eight recently released crystal structures of PKMYT1, focused on docking (GOLD [69]) and binding free energy analysis (AMBER [70]). Facing difficulties in unambiguously defining the binding mode of a new diaminopyrimidine chemotype, they undertook an indirect validation of docking poses by looking for highly similar small molecules cocrystallized with other kinases. This was achieved through dual scoring LBVS towards the LigandExpo collection [46] with SwissSimilarity. As a result, 23 similar cocrystallized ligands showing sufficient 2D and 3D similarity were found. Emphasis was given to thoroughly analyzing aminopyrimidine inhibitors of Aurora A kinase, for which the experimental binding modes in several structures [71,72] were very close to the ones predicted in PKMYT1. These dependable docking models, validated through chemical and shape similarity screening, allowed them to start an in silico optimization process through QSAR and fragment-growing strategies, leading to a series of inhibitors active on PKMYT1 at the submicromolar level and to a solid rational basis to design more potent analogues.

Chemical and shape similarity approaches were employed by Bhunia, D. et al. for the development of short non-toxic cell-penetrating peptides [73]. For the design of the most promising tetrapeptide (ETWW), mainly driven by the spatial position of tryptophan residues, determining the cell surface receptors responsible for cellular uptake was crucial and tackled by a LBVS strategy. ETWW was submitted to SwissSimilarity and SwissTargetPrediction 2D-/3D-dual similarity searches. The outcome of both the direct and the reverse screenings yielded diverse small organic molecules highly similar in structure and shape to the tetrapeptide and known ligands of two important cell membrane receptors. Moreover, the probability quantified by SwissTargetPrediction gave confidence to the authors to select the endothelin B and the mu opioid receptors for docking studies and finally for experimental endocytosis studies. The results validated the hypothesized mechanism. Additionally, two pharmacokinetics classification models implemented in SwissADME [16] were employed to evaluate if the peptide is sensitive to cell efflux and to metabolic liability. The first classifier enables to predict if a molecule is prone to be a substrate of the permeability glycoprotein, abbreviated P-gp, and responsible for the most important active efflux mechanism of xenobiotics through biological membranes [74]. A support vector machine (SVM) was trained on 521 P-gp substrates and 512 non-substrates to achieve a classification accuracy of 89% on an external test set of 415 compounds. Five other SVM-based models regard the five most important isoenzymes of cytochromes P450. These were trained and tested on thousands of different molecules and achieved external accuracy of 91%, 87%, 81%, 87% and 86% for CYP1A2, CYP2C19, CYP2C9, CYP2D6 and CYP3A4, respectively [16]. With these robust predictive models, Bhunia, D. et al. estimated that ETWW is not a substrate of the P-glycoprotein efflux pump, nor of CYP450 metabolizing enzymes. These predictions were experimentally confirmed and established the ETWW tetrapeptide as a promising drug-delivery vehicle.

As mentioned above, SwissDock was developed for accurate physics-based analysis of ligand-protein binding and therefore is not fast enough to perform VS on large chemical libraries [5]. It was nevertheless found to be of great value for the confirmation of prior fast dockings, in a two-step SBVS process. An example is a drug discovery study targeting a p53 mutant, present in many cancer tissues and responsible for malignant tumor progression [75].

A subset of ZINC [34] with more than 800,000 drug-like compounds was docked into an allosteric site of the crystal structure of the p53 mutant R273H using the idock fast docking web service (istar.cse.cuhk.edu.hk/idock). Top-scored molecules were re-docked into the same tridimensional structure with SwissDock. Based on calculated binding free energies and scaffold diversity, 12 compounds were selected and their activity was experimentally validated in cell-based experiments on wild-type and mutant p53. The results demonstrate the potential of diverse chemotypes to become interesting candidates as mutant p53 reactivating agents.

## 4. Conclusions

Computer-aided drug design methods can support experimental screening of small molecules in several ways. Accordingly, the methods included in the SwissDrugDesign project have an important role to play in various drug discovery strategies, as exemplified in this short review. Besides ligand-based methods especially developed for virtual screening, such as SwissSimilarity and SwissTargetPrediction, other tools dedicated, for instance, to molecular docking (SwissDock) or pharmacokinetics (SwissADME) can also participate in efficiently enriching small molecule libraries. Being web based, cost- and login-free, these tools are particularity well suited for academic and non-for-profit screening campaigns. As illustrated here, the described web-based tools have already played a role in the discovery of bioactive compounds, confirming their effectiveness, and beyond that, the general usefulness of CADD tools. The application examples also show that, in certain circumstances, non-experts can take advantage of advanced in silico methods through simplified interfaces to obtain relevant and experimentally validated results. Extending the usage of CADD within the life science community is indeed one of the major objectives of the SwissDrugDesign project.
